# The Story of the Insane from Year to Year

**Published:** 1904-02-06

**Authors:** 


### THE STORY OF THE INSANE FROM YEAR TO YEAR.

Sixteenth Annual Series.

### GLAMORGAN COUNTY ASYLUM.

On the first of January the numbers in residence were 1,841 and at the end of the year 1,910. There were 482 first admissions, 80 readmissions, 165 recoveries, 37 discharged relieved, 110 discharged not improved, and 181 died. The average number resident was 1,864, and the total number under treatment was 2,395. Calculated on the admissions the recoveries stand at 31·4 per cent., and calculated on the average number resident the deaths were 9·7 per cent. Of the year's deaths 62 were caused by cerebral and spinal diseases (34 of these being general paralysis), 20 by consumption, 6 by other lung diseases, 2 by enteritis, and 1 by scarlatina. Various moral causes are supposed to account for the insanity in 142 of the admissions, and among the physical causes intemperance in drink accounts for 133 and hereditary influence for 137. It is thus seen that more than 50 per cent. of the insanity of Glamorgan is caused by these two preventible causes, drink and heredity. The asylum is greatly overcrowded, and although the new block will have been opened before this is in type, there will still be 115 more patients than the asylum is designed to accommodate. It is therefore proposed to dissolve the union between the County of Glamorgan and the Borough of Swansea. The death-rate from tubercular disease was 13 per cent. of the total death-rate, and it would seem desirable, especially as room is so much wanted, to run up an annexe for the separate treatment of consumption. There are six medical officers and three chaplains on the staff, so that both spiritually and physically the patients should be well looked after. The committee record "their high appreciation of the exceptional skill, rare administrative ability, and uniform kindness exhibited by the medical superintendent." The weekly charge to the Unions is 9s. 4d. per head.

